# Impact of Nutrition and Salinity Changes on Biological Performances of Green and White Sturgeon

**DOI:** 10.1371/journal.pone.0122029

**Published:** 2015-04-01

**Authors:** Pedro G. Vaz, Ermias Kebreab, Silas S. O. Hung, James G. Fadel, Seunghyung Lee, Nann A. Fangue

**Affiliations:** 1 Department of Animal Science, University of California Davis, Davis, California, United States of America; 2 Department of Wildlife, Fish and Conservation Biology, University of California Davis, Davis, California, United States of America; Virginia Commonwealth Univ, UNITED STATES

## Abstract

Green and white sturgeon are species of high conservational and economic interest, particularly in the San Francisco Bay Delta (SFBD) for which significant climate change-derived alterations in salinity and nutritional patterns are forecasted. Although there is paucity of information, it is critical to test the network of biological responses underlying the capacity of animals to tolerate current environmental changes. Through nutrition and salinity challenges, climate change will likely have more physiological effect on young sturgeon stages, which in turn may affect growth performance. In this study, the two species were challenged in a multiple-factor experimental setting, first to levels of feeding rate, and then to salinity levels for different time periods. Data analysis included generalized additive models to select predictors of growth performance (measured by condition factor) among the environmental stressors considered and a suite of physiological variables. Using structural equation modeling, a path diagram is proposed to quantify the main linkages among nutrition status, salinity, osmoregulation variables, and growth performances. Three major trends were anticipated for the growth performance of green and white sturgeon in the juvenile stage in the SFBD: (i) a decrease in prey abundance will be highly detrimental for the growth of both species; (ii) an acute increase in salinity within the limits studied can be tolerated by both species but possibly the energy spent in osmoregulation may affect green sturgeon growth within the time window assessed; (iii) the mechanism of synergistic effects of nutrition and salinity changes will be more complex in green sturgeon, with condition factor responding nonlinearly to interactions of salinity and nutrition status or time of salinity exposure. Green sturgeon merits special scientific attention and conservation effort to offset the effects of feed restriction and salinity as key environmental stressors in the SFBD.

## Introduction

As global change biologists forecast the impacts of global climate change on contemporary species, consideration of interactive and possibly synergistic stressors is of critical importance [1–2]. Current predictions include temperature rise [3] accompanied by increases in sea level [4], followed by increments in salinity across some estuaries worldwide [5]. The distribution and abundance of organisms will shift according to their thermal tolerance limits and adjustment ability, with the consequent changes through the trophic cascade [6–9]. Among species of special concern [10–11] in estuaries, green sturgeon (*Acipenser medirostris*) and white sturgeon (*A*. *transmontanus*) will likely be affected by climate change derived alterations such as salinity, and prey type and abundance. In addition, nutrient availability and composition may be affected in water bodies. To date, there has not been a systematic study designed to establish the relationship between nutritional status, as an indicator of dietary quality and quantity, and growth and physiological performances of green and white sturgeon, when faced with salinity changes. This study takes advantage of relatively new modelling techniques [12–14] to test the network of direct and indirect causal relationships among these climate change derived stressors, physiological variables, and the growth performance of these two species of high conservational, recreational, and economic interest.

Significant changes in salinity and nutritional patterns are forecasted across the distribution area of green sturgeon (Mexico to the Bering Sea), and white sturgeon (Mexico to the Gulf of Alaska), particularly in estuary areas [15]. The San Francisco Bay Delta (SFBD) system is particularly relevant because both species of sturgeon are native and likely to be most impacted by global change. The SFBD is greatly affected by changes in oceanic conditions with a multitude of well-documented abiotic and biotic changes occurring over a variety of time scales [16–17]. The effects of global climate change in SFBD include increasing salinity as a result of sea level rise and seawater intrusion into the Delta, changes in precipitation patterns, and a smaller snowpack, contributing to a lower spring freshwater runoff and nutrients. Of particular importance to sturgeon, salinity is projected to change in magnitude, timing, and space in the SFBD [18–21].

In addition, food webs are changing globally and locally. Increased water temperature decreases global phytoplankton production at a projected rate of 1% per year [22] disturbing the synchrony between phytoplankton and zooplankton [23]. Food web dynamics in the Pacific Ocean and SFBD have been under extensive transformation over the past few decades due to shifts in phytoplankton and zooplankton communities as well as an increase in exotic species dominance. While food webs in the SFBD system are changing [24–26], sturgeon diets can shift to reflect availability and abundance of prey. The recent tendency of white sturgeon consuming mainly clam species introduced to the west coast of the USA is an example of this [27].

Climate change may have physiological and biochemical effects on sturgeon through nutrition and salinity challenges, which in turn reduces growth performance or fitness [28]. Assessing physiological tolerance ranges and thresholds to stressors may determine whether sturgeon have enough resilience to respond to climate change [29]. Emphasis has to be placed on early animal life-stages, which may be more sensitive to environmental stressors than adults [30]. Young sturgeon stages are poorly understood and the success of young fish as they move through the SFBD is likely dependent on their nutritional status and on the timing of development of physiological mechanisms matched to their migration habitats.

Previous studies have examined nutrition status effect on some osmoregulation variables in fish. For example, Atlantic salmon (*Salmo salar*) exhibited an increase in plasma ions and reduction in enzymatic activities following six- and eight-week food deprivation periods [31–32], whereas coho salmon (*Oncorhynchus kisutch*) and chinook salmon (*O*. *tshawytscha*) osmoregulation were not affected after 16 weeks of food deprivation [33]. Osmoregulation responses have also been examined in sturgeon species [34–44], suggesting that metabolic cost of osmoregulation can vary with salinity, especially during juvenile stages [45–47]. Recently, the effect of nutritional status on several osmoregulation variables in juvenile green sturgeon were evaluated [48]. To date considerable progress in determining single responses of physiological variables to nutritional status and salinity has been achieved.

Moving beyond single-variable responses, it is critical to determine the mechanisms underlying the capacity of animals to tolerate environmental changes [49]. Moreover, there are species-specific responses that must be considered, even when assessing closely related species, like green and white sturgeon. In this study, growth performance of juvenile green and white sturgeon were assessed following nutrition and salinity changes, elucidating cause-and-effect relationships among these key-changes and a suite of biological responses. The two sturgeon species were challenged in a multiple-factor experimental setting, first to levels of feeding rate, and then to salinity levels for different time periods. The study aimed to: (i) compare juvenile green and white sturgeon in their growth performance, body composition and plasma metabolites; (ii) assess the effect of feeding rate, species, and their interaction on the biological variables; (iii) select predictors of growth performance in juvenile green and white sturgeon following feeding rate and salinity changes; and (iv) quantify how nutrition status, salinity, and osmoregulation interact to influence growth performance in both species. The study hypothesized that: (i) the biological performance (growth, body composition, plasma metabolites) would differ in juvenile green and white sturgeon with the same nutritional status; (ii) growth performance in juvenile green and white sturgeon would be determined by different factors regarding nutrition and salinity changes, and osmoregulation variables; (iii) besides direct effects, nutrition and salinity would affect growth performance in both species indirectly through relationships with osmoregulation variables.

## Materials and Methods

### Fish source

Juvenile green sturgeon were obtained from captive F1 broodstock, reared from wild-caught Klamath River and they were spawned in 1999–2000 [50] and held in the Center for Aquatic Biology and Aquaculture at the University of California, Davis, USA. The female (1999 year class) was tank spawned with two males from the 2000 year class [51]. Juvenile white sturgeon were donated by a local fish farmer who spawned them from one domesticated female (~46 kg, 12 years old) and four domesticated males (~28 kg, 8 years). The progenies of the two species were reared in two flow through systems of degassed ground water and fed the same commercial salmonid starter diet including a variety of salmonid feeds until they reached a desired size for the experiment. The two species were fed according to a model of optimal feeding rate (OFR) for green [52] and white [53] sturgeon.

### First phase—nutrition challenge

**Fig 1:**
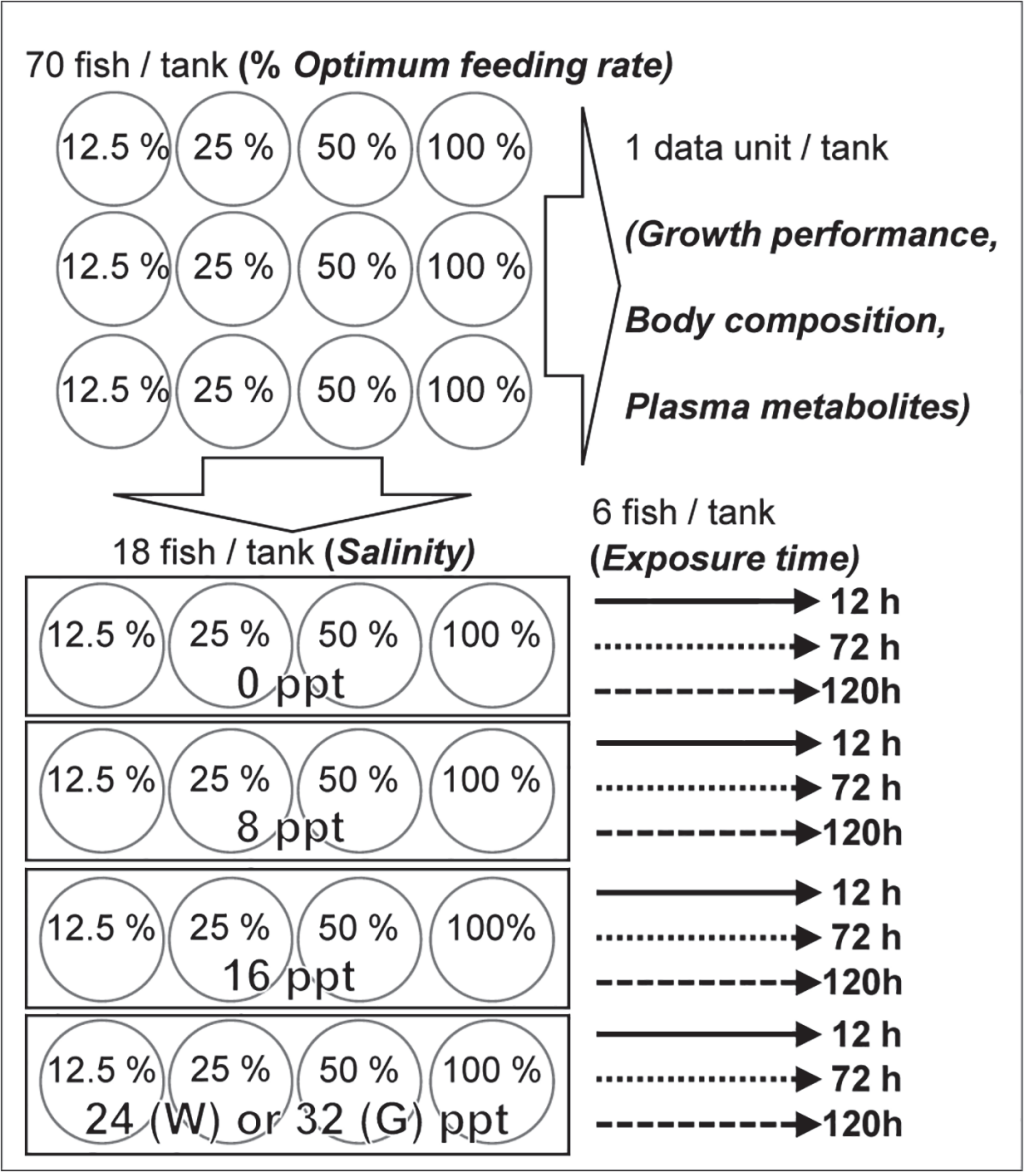
Experimental design. First phase (top): Four levels of one treatment, feeding rate (12.5, 25, 50, 100%). Second phase (bottom): Four levels of the treatment salinity (0, 8, 16, 24/32 ppt), and three levels of the exposure time to salinity levels (12, 72, 120 h). The experimental design was the same for the two species, except that maximum salinity was different for white (W) and green (G) sturgeon.

This experiment phase was initiated at 214 and 189 days post hatch in green and white sturgeon, respectively, and was replicated similarly for the two species. For each species, 840 juvenile sturgeons (green: 174.0 ± 0.4 g, white: 173.2 ± 0.6 g; mean ± SE) were randomly chosen and released into 12 circular, flow through fiberglass ~787 L tanks, resulting in 70 fish per tank (Fig 1). Fish were acclimatized to the tanks for 8 days and fed at the OFR (2.0 mm sinking pellet, Skretting, Tooele, UT, USA). Feeds were given using a 24-h belt feeder to ensure continuous food availability [54]. Holding tanks were located outdoors and had a fiberglass cover with a hatch allowing access of feed and sunlight of natural photoperiod. An angled spray-bar supplied degassed well water (8–10 L min^-1^) to increase circulation and feed dispersion. Water temperature (18.1 to 18.7°C), dissolved oxygen (7.5 to 9.0 mg L^-1^), and ammonia (0.1 to 0.2 mg L^-1^) were maintained throughout the trial.

Upon the end of the acclimatization period, the average initial body weight (BW_i_) per tank of sturgeon were measured to adjust feed quantity prior to initiation of a four-week nutrition challenge trial. Twelve experimental tanks per species were then randomly assigned to one of the four levels of feeding rate (FR) treatment (12.5%, 25%, 50%, 100% of OFR; [53, 55]), resulting in three tanks per treatment per species (Fig 1, top). Diet proximate compositions for the acclimatization period and the nutrition challenge trial were 8.7% moisture, 42% crude protein, 26.7% crude lipids and 9.9% ash, as determined by the Association of Official Analytical Chemists method (AOAC; [56]).

After the four-week nutrition challenge, growth performance variables, body composition, and plasma metabolites were determined as follows. One data unit per variable was determined for each tank. First, final body weight (BW_f_) per tank was derived from the average of all the fish in the tank. Fish total length (cm) and liver weight (g) were determined (precision: ± 0.01, in both measurements) from six fish per tank that were euthanized.

Growth performance metrics were calculated as:

- Specific growth rate (SGR) = 100 × (ln (BW_f_ /BW_i_) /D_t_);
- Feed efficiency (FE) = 100 × (BW_f_—BW_i_) /F_t_; where BW_i_ and BW_f_ were the average initial and final body weight (g), D_t_ was trial length in days (26), and F_t_ was total average weight of feed (g) given to each tank throughout the trial.
- Condition factor (CF) = BW_f_ / L^3^; where L was total body length (cm);
- Hepato-somatic index (HSI) = 100 × liver weight (g) /BW_f_; calculated after fish euthanasia.

Body composition and plasma metabolites following the nutrition challenge were determined from the six fish euthanized per tank. Three of these fish were pooled and sampled for whole body proximate composition (AOAC method, [56]) to determine crude lipid, crude protein, and moisture. The remaining three fish were sampled individually for assessment of plasma metabolites (glucose, triglycerides, and protein) concentrations followed by an average calculation per metabolite. Blood was collected from the caudal vein using a 6 ml blood collection tube with dry lithium heparin and a 21-gauge hypodermic needle and was centrifuged at 1500 *g* for five minutes at room temperature. Plasma was transferred to 1.5 m1 micro-centrifuge tubes, snap frozen in liquid nitrogen and stored at -80°C for later analysis. Plasma glucose concentrations were determined with a commercially available assay kit. Plasma triglycerides were measured by a quantitative enzymatic measurement using a serum triglyceride determination kit (Sigma Aldrich, St. Louis, MO, USA). Plasma total protein concentration was determined by the Sigma Aldrich Micro-Lowry, Onishi & Barr modification method.

### Second phase—Salinity challenge

Following the nutrition challenge trial, a salinity challenge with different exposure times was subsequently conducted and replicated similarly for green and white sturgeon (Fig 1 bottom). Four salinities were selected for both species trials, differing in the highest level only, *i*.*e*., 0, 8, and 16 ppt for both species, and 24 or 32 ppt for white and green sturgeon, respectively. The salinity levels were selected on the basis of life histories of juvenile green and white sturgeon [57–59]. The four salinity levels were applied in four separate systems for each species, each consisting of four tanks (97 cm diameter, 160 L): one flow-through freshwater system (0 ppt), and three separate recirculating systems (8, 16, and 32 or 24 ppt) where salinity was manipulated using synthetic sea salt.

Each of the four tanks per salinity level was then assigned to one of four FR (12.5%, 25%, 50%, or 100% of OFR) and occupied with 18 fish from that FR (Fig 1 bottom). Fish were acutely exposed to different salinity levels and were not fed for one day prior to salinity exposure and throughout the trial. Water quality (*e*.*g*., temperature, dissolved oxygen, ammonia) was maintained at optimum conditions during the trial. Osmoregulation variables, including pyloric caeca and gill Na^+^/K^+^-ATPase activities (PCNKA, GNKA, respectively), muscle moisture, hematocrits, plasma osmolality, lactate, and glucose were then determined at 12, 72, and 120 h following the salinity exposure (Fig 1 bottom). Additional details (*e*.*g*., analytical procedures) are provided in [48].

### Ethics Statement

This study was carried out in strict accordance with the recommendations in the protocol approved by the Campus Animal Care and Use Committee of the University of California, Davis (Protocol Number: 16541). The Committee approved this study and verified that the living conditions of the animals were appropriate for the species, that the use of pain-relieving drugs is adequate, and that the number of animals was the minimum necessary to complete the project. Fish euthanasia was performed under an overdose of buffered MS-222 (6 g NaCl, 420 mg NaHCO3 and 500 mg tricaine methanesulfonate/L, Argent Inc., Redmond, WA, USA).

## Data analysis

### Biological responses to nutritional changes

For comparison of the biological responses in green and white sturgeon to nutritional changes, the metric for each variable was standardized between 0 and 1 using the equation *v’ = (v*_*i*_ *—v*_*min*_ *) /(v*_*max*_ *—v*_*min*_ *)*; where *v*_*i*_ is each value (untransformed) and *v*_*min*_ and *v*_*max*_ are the minimum and maximum for that variable.

Before analysis, some variables were transformed to approach normality and homoscedasticity assumptions. HSI, lipids, body protein, and moisture were arcsin√(*x*)-transformed. Using the equation *v’ = (v*_*i*_^*λ*^ *-1)/λ*, the Box-Cox family of transformations was used on each *i* ^th^ value to find the best transformation [60–61] on glucose (λ = -1.43), triglycerides (λ = 0.02), and protein (λ = -1.55). Main effects and interactions of FR and species on SGR and CF were tested using one two-factor ANOVA per response. A permutation version of the test with 5000 randomizations was conducted for other responses, using lmPerm package in R [62].

### Predictors of growth performance in sturgeons faced with nutritional and salinity changes

Condition factor was used as the response for two separate models on the effect of salinity on growth performance for the two species. Calculated from the relationship between weight and length, CF is widely used in fisheries and fish biology studies as an excellent indicator of the degree of food sources availability and general well-being [63–64]. The relationship between CF and explanatory variables was explored using Generalized Additive Models (GAM; [65–66]) with identity link to account for potential non-linearities in CF responses. A few clear outliers were determined and omitted after initial exploratory data analysis using boxplots and Cleveland dotplots [67]. For green sturgeon, five outliers were omitted, three for CF and two for muscle moisture (N = 277). For white sturgeon, 12 outliers were omitted for CF (N = 276). Spearman’s correlations between the variables in the models were all < |0.30|, indicating that there were no co-linearity problems. Prior to statistical analysis, PCNKA and GNKA were log-transformed to approach normality and to reduce the influence of a few large values. For the analysis, FR, time, and salinity were treated as factors. All calculations were carried out using R [68]. The mgcv package [65] was used to fit GAM, using penalized regression splines with the optimal amount of smoothing estimated by generalized cross validation (GVC).

For both species of sturgeon, a full model of GAM was fitted with ten variables, *i*.*e*., FR, salinity, time, PCNKA, GNKA, moisture, lactate, osmolality, glucose, and hematocrit. The full model included FR × time × salinity interactions, followed by backward elimination of non-significant (*p* > 0.05) variables [66] to remove each main term in turn. The significance of each parametric and smooth term was assessed using Wald like tests (mgcv package; [69–70]). Model adequacy was evaluated by plotting residuals vs fitted values and explanatory variables and model fit by the percentage of the deviance explained.

### Linkages between best predictors of growth performance

Structural equation modeling (SEM; [71–72]) was used to separately examine the linkages between significant terms in GAM related to CF. Additionally, SEM highlighted indirect effects not revealed by GAM. Using the software IBM SPSS Amos, a path diagram was constructed first based on theory using the exogenous variables for CF. Error terms were added as needed [71], and regression weights were examined to iteratively add (based on modification indices) or remove (based on *p*-values) linkages from the model. Once a good model fit was achieved, based on both the minimum discrepancy [73] and the root mean square error of approximation, CF was added as an endogenous variable with linkages from all other variables. Bayesian estimation was then used on the retained paths to fit the model, and linkages to CF were iteratively removed based on the posterior distributions of the regression weights. Linkages were removed if their 80% credible interval included zero (considered supportive of a model derived from maximum likelihood procedures).

## Results

### Biological responses of green and white sturgeon to nutritional changes

**Fig 2:**
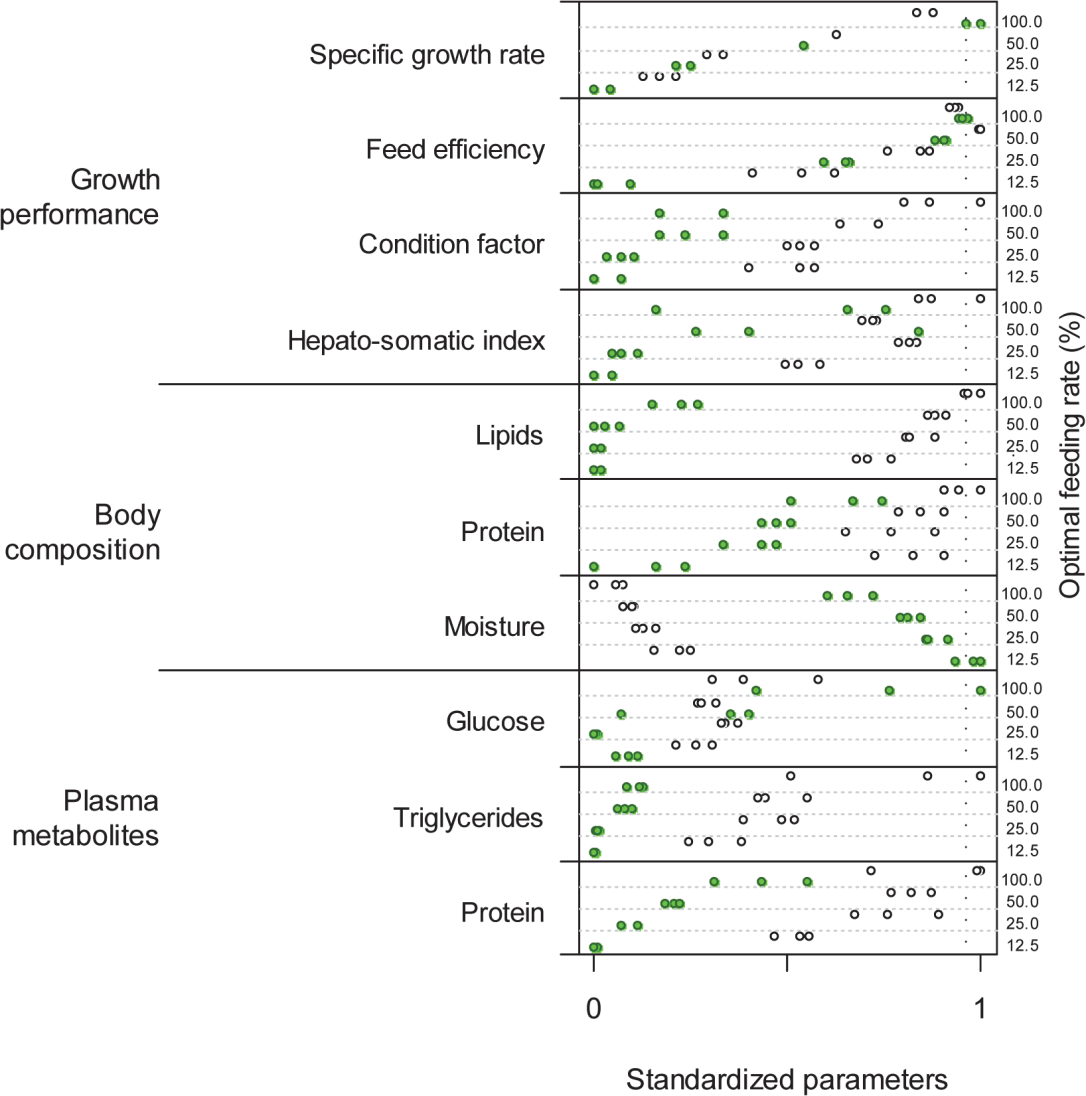
Standardized values (0 to 1) of biological responses to nutritional changes. Green and white dots denote green and white sturgeon, respectively. For each biological parameter (y-axis), fishes were challenged with four levels of feeding rate (12.5, 25, 50, 100%; right y-axis). Three observations were collected per FR level per species.

Green and white sturgeon had in general contrasting results concerning measured factors of growth performance, body composition, and plasma metabolites (Fig 2). Body moisture was the only variable clearly greater for green than for white sturgeon independent of feeding rate. Regarding growth performance, CF was the response without any overlap between species across FR levels.

For all biological variables, the effect of FR was significant and the response of the two species differed (Table 1). Moreover, the FR × species interaction was not significant only for CF, indicating that the effect of FR on CF was probably comparable as it followed a similar pattern for green and white sturgeon across FR levels. At 12.5 and 25% of OFR, white sturgeon always showed better biological performance for all the variables. At 50 and 100% of OFR, some overlap was found between species for several biological variables. Noticeably, at 100% of OFR, mean performance of green sturgeon was greater regarding specific growth rate and feed efficiency (Fig 2). During the nutrition challenge three fish mortalities occurred for green sturgeon over the fourth week, one at 12.5% and two at 50% of OFR.

**Table 1:** Significance levels of main effects and interactions for feeding rate and species on biological responses.

		Factors		
Variable	Response	Feeding rate	Species	Feeding rate × Species
Growth performance	Specific growth rate	***	***	***
	Feed efficiency	***	***	***
	Condition factor	***	***	NS
	Hepato-somatic index	**	***	*
Body composition	Lipids	***	***	**
	Body protein	***	***	**
	Moisture	***	***	**
Plasma metabolites	Glucose	***	***	***
	Triglycerides	***	***	***
	Plasma protein	***	***	**

For specific growth rate and condition factor ANOVA were conducted whereas for other responses p-values were generated from permutation ANOVA analyses.

*** ≤ 0.001

** ≤ 0.01

* = 0.05

NS ≥ 0.05.

### Predictors of growth performance in green and white sturgeon faced with nutrition and salinity challenges

**Fig 3:**
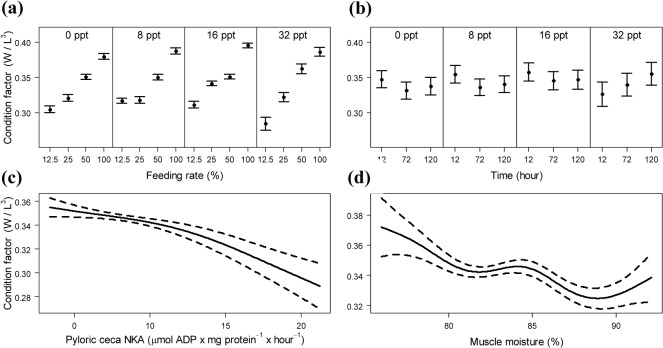
Generalized additive model fits and 95% confidence intervals from the optimal model for green sturgeon. Relationships between growth performance of juvenile green sturgeon, measured by condition factor, and each explanatory variable (FR × salinity interaction, time × salinity interaction, pyloric ceca NKA, and muscle moisture). Sturgeons were challenged in a multiple-factor setting, first to levels of feeding rate (12.5, 25, 50, 100%), and then to salinity levels (0, 8, 16, 32 ppt) for a given time (12, 72, 120 h). W = weight; L = length.

In green sturgeon, evaluation of the significance of predictors of CF as an indicator of growth performance resulted in the sequential dropping of FR × time × salinity interaction, FR × time interaction, lactate, GNKA, glucose, hematocrit, and osmolality. The resulting model included FR × salinity and time × salinity interactions, along with PCNKA and muscle moisture (Table 2). The effect of salinity on CF was therefore not the same for all feeding rates or exposure times (Fig 3). Fitted values of CF tended to be higher as FR increases, except that for 8 ppt salinity level CF values were similar at 12.5% and 25% of OFR (Fig 3A). Condition factor did not differ among exposure times to salinity but mean fitted CF at 12 h was lower than for longer exposures (72, 120 h) at 32 ppt salinity only (Fig 3B). Pyloric caeca NKA enzyme activities greater than ~10 μmol ADP mg protein^-1^ h^-1^ tended to be associated with a steeper decrease in CF (Fig 3C). Also, as muscle moisture increased, CF showed a non-linear decreasing trend (Fig 3D).

**Table 2:** Significance of final models’ terms.

**Green sturgeon**	**(DE = 63.20%; GCV = 0.001)**		
Parametric terms	df	*F*	*p*
*FR*	3	13.40	<0.0001
*Salinity*	3	2.95	0.033
*Time*	2	2.99	0.052
*FR × Salinity*	9	2.09	0.030
*Time × Salinity*	6	3.58	0.002
Smooth terms	edf	*F*	*p*
*Pyloric ceca NKA*	1.969	3.37	0.027
*Muscle moisture*	4.716	2.17	0.041
**White sturgeon**	**(DE = 32.20%; GCV = 0.002)**		
Parametric terms	df	*F*	*p*
*FR*	3	40.22	<0.0001
*Time*	2	3.77	0.024

The two models explain growth performance measured by condition factor in juvenile green and white sturgeons challenged in a multiple-factor setting with levels of feeding rate (FR; 12.5, 25, 50, 100%), salinity (0, 8, 16, 24 ppt for white and 32 ppt green sturgeon), and time of exposure to salinity (12, 72, 120 h). Seven osmoregulation parameters were tested as covariates. DE = deviance explained; GCV = generalized cross validation score; edf = effective degrees of freedom.

**Fig 4:**
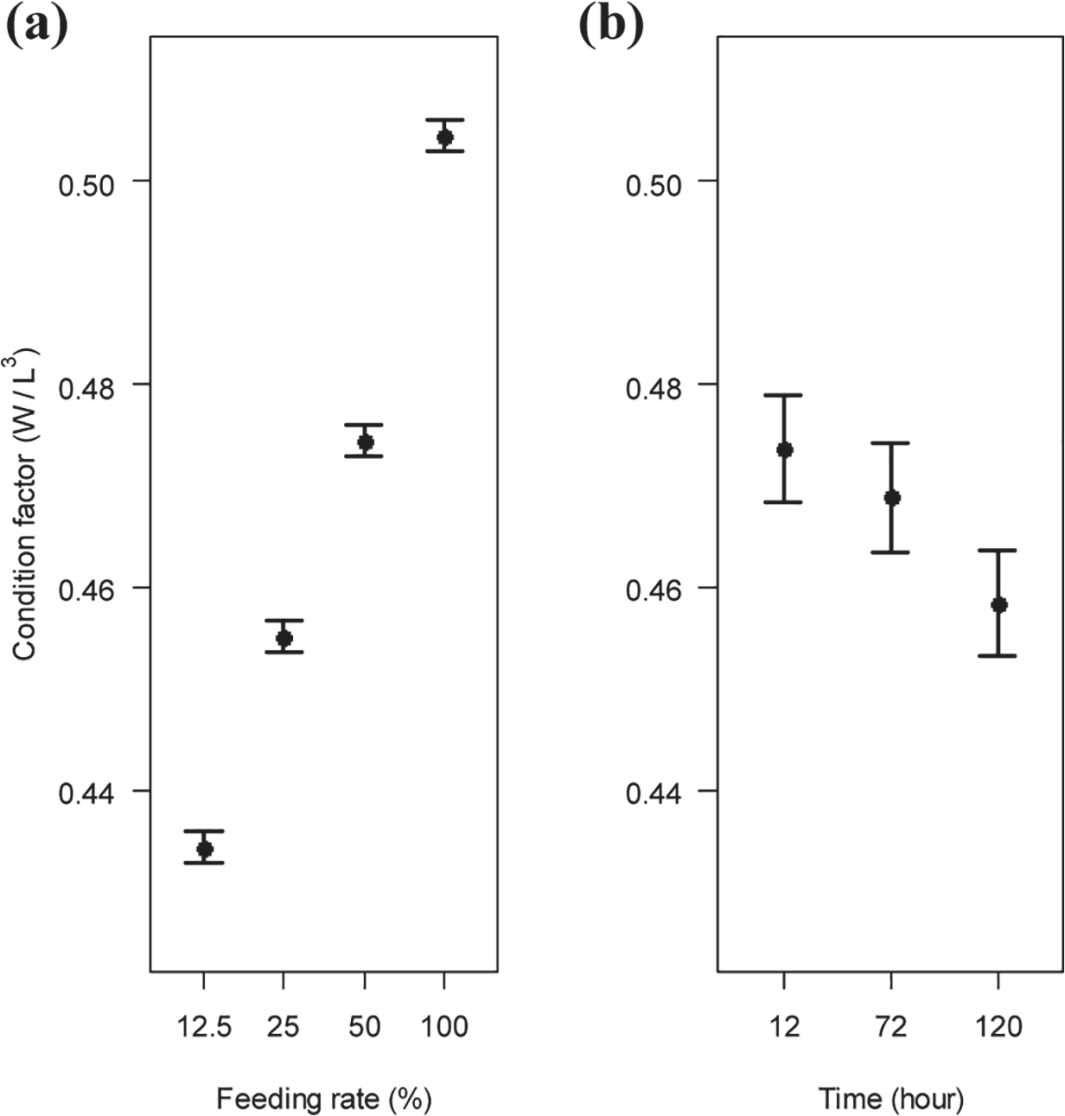
Generalized additive model fits and 95% confidence intervals from the optimal model for white sturgeon. Relationships between growth performance, measured by condition factor, and each explanatory variable are illustrated. Sturgeons were challenged in a multiple-factor setting, first to levels of feeding rate (12.5, 25, 50, 100%), and then to salinity levels (0, 8, 16, 24 ppt) for a given time (12, 72, 120 h). Only feeding rate and time were kept in the final model. W = weight; L = length.

In white sturgeon, all treatments’ (FR, salinity, time) interactions were removed in turn, followed by PCNKA, salinity, glucose, hematocrit, muscle moisture, and lactate. The final model included FR and time of exposure to salinity only as the best predictors of CF (Table 2). Condition factor increased with increments in FR level (Fig 4A). Conversely, CF fitted values were greater for fish exposed to salinity levels for 12 h than over 120 h, whereas the 72 h effect on CF was intermediate (Fig 4B). During the salinity challenge 13 fish mortalities occurred for green sturgeon at 72h salinity exposure, seven at 12.5%, five at 25%, and one at 50% of OFR.

### Linkages between best predictors of growth performance using SEM

**Fig 5:**
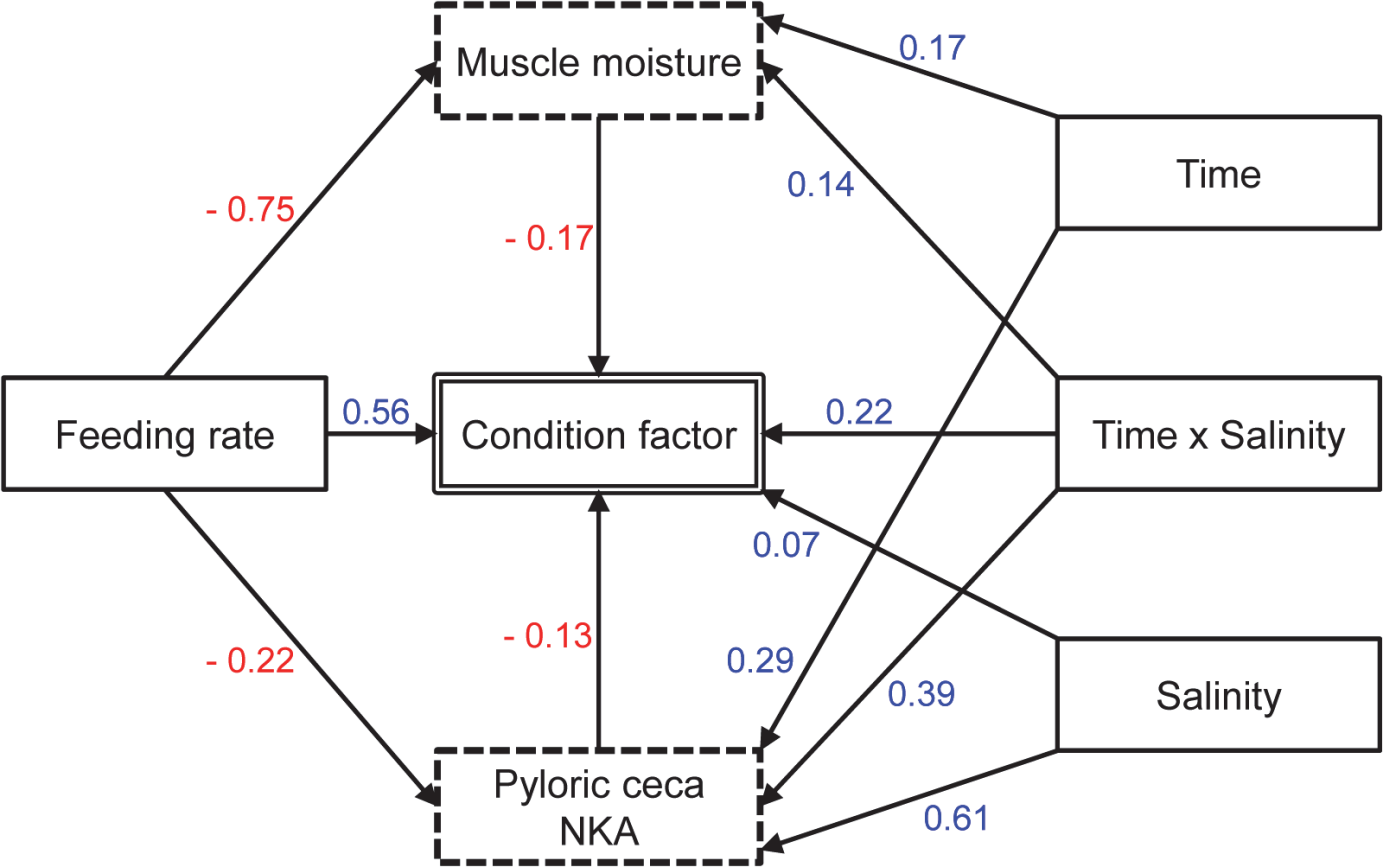
Fitted structural equation model for green sturgeon. Bayesian estimation was used to determine variables affecting condition factor (double-line rectangle). Solid rectangles: treatments; dashed rectangles: osmoregulation parameters. Arrows represent causal pathways between variables, each having a standardized partial regression coefficient (sign indicates whether the relationship is positive or negative for that direct effect).

A variety of linkages were present among significant predictors of CF in juvenile green sturgeon (Fig 5). The terms that significantly affected CF were the same as those identified by the GAM, except that FR × salinity interaction was dropped based on the posterior distribution of its regression weight. Feeding rate had the greatest direct effect on CF. In addition to direct effects, several indirect effects (those connecting predictor variables) were identified where the variable’s effect on CF was mediated by another variable. Among indirect effects, feeding rate-muscle moisture and salinity-PCNKA were clearly the strongest linkages, with regression weights > |0.60|. All linkages retained in the model had coefficients with a 95% credible interval, except salinity-CF for which the cutoff of 80% was used. The final SEM had a posterior predictive p = 0.55, indicating a good fit [12]. The SEM was advantageous in identifying both unique and synergistic contributions of CF predictor variables. For white sturgeon, because only FR and time were selected by GAM as significant predictors of CF, SEM was not used.

## Discussion

**Fig 6:**
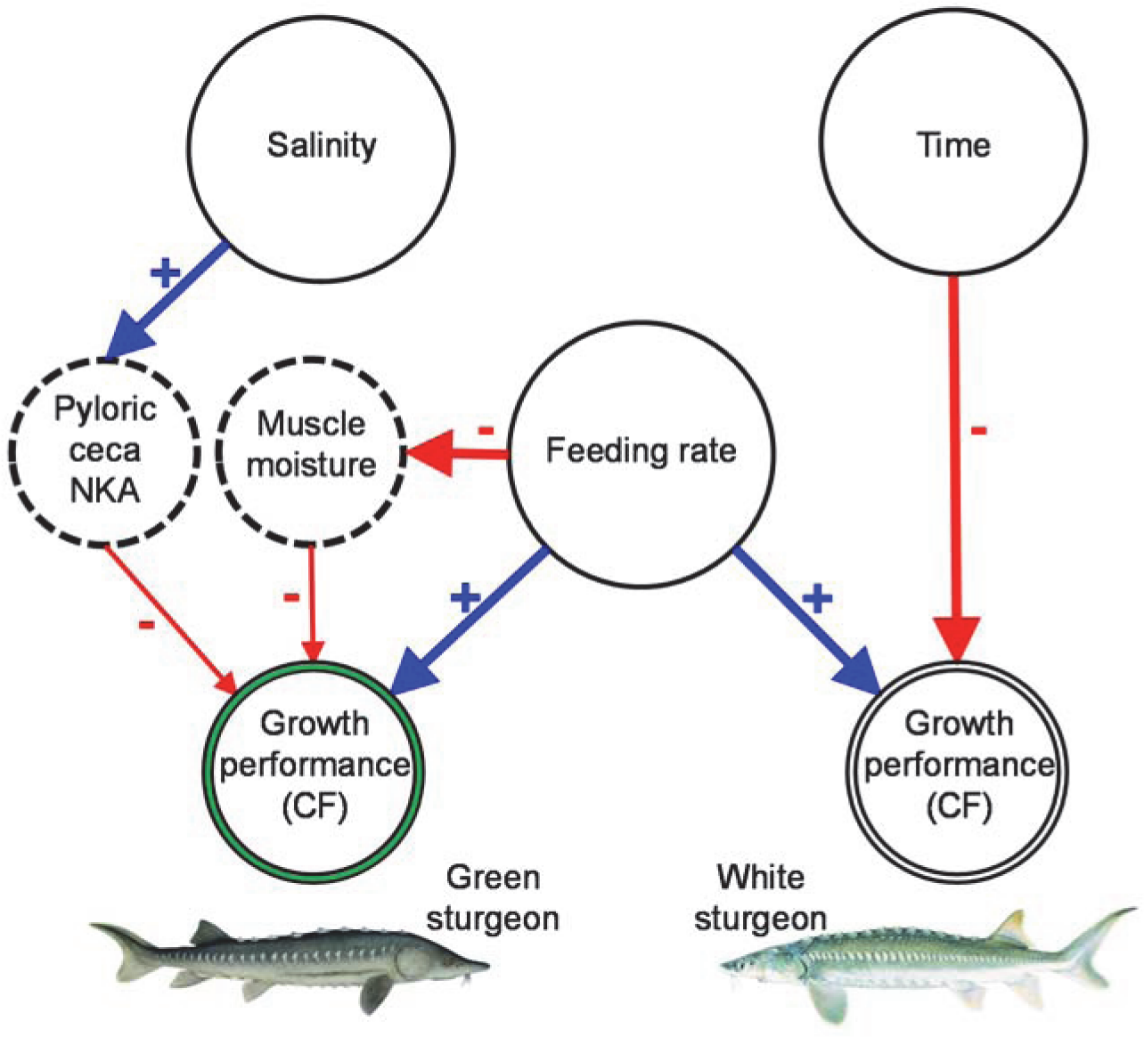
Schematic overview of the primary results. The main relationships among the principal variables identified in this study are summarized as affecting directly or indirectly the growth performance of green and white juvenile sturgeon when challenged with levels of their feeding rate, salinity, and exposure time to salinity. Solid circles: treatments; dashed circles: osmoregulation parameters; double-line circles: growth performance measured by condition factor (CF). The sign associated with each arrow indicates whether the relationship is positive or negative for that effect. Wider arrows refer to the most significant relationships in this study.

Recently, progress has been made in determining individual responses of biological variables in fish to climate change derived stressors. Broadly, the contribution of animal physiology in the study of global climate change has been highlighted [74–77] unveiling physiological thresholds and tipping points. Clear links have been established between changes seen at the ecosystem level and physiological limitations detected through well-controlled laboratory experiments [41, 78]. For example, it has been recently shown that exposure of a variety of aquatic species to climate change relevant stressors results in dramatic changes at the biochemical level but also subtle changes in growth patterns, many of which were not initially predictable from whole organism studies [79–81]. However, most studies do not include network analysis of direct and indirect causal relationships among environmental stressors, physiological variables, and growth performance. In this study, a summarized path diagram is proposed to quantify the main linkages among nutrition status, salinity, main osmoregulation variables, and growth performances of juvenile green and white sturgeon assessed by CF (Fig 6). As hypothesized, besides direct effects, nutrition and salinity affected growth performance indirectly through relationships with osmoregulation variables. Furthermore, comparison of the species showed that the linkages were species-specific. The differences may explain why these two species, in spite of their sympatry and taxonomic proximity, may respond differently to potentially novel environmental stressors caused by global change.

### Impact of nutrition change on biological performance of juvenile sturgeon

Overall, feeding rate was the most relevant factor affecting the biological responses considered, whether after the nutritional challenge or following the salinity challenge. To date, individual response of several aspects of biological performance in fish to nutrition status have been widely studied [31, 53–54, 82–86]. In all studies, feed restriction *per se* proved to be a crucial stressor influencing directly many responses of fish ecophysiology. This study followed the same trend, in which feeding rate appeared to be a determinant for all 10 biological variables considered after the nutritional challenge. Moreover, the first hypothesis that growth performance, body composition, and plasma metabolites would differ between the two sturgeon species with the same nutritional status was in general supported. Furthermore, the effects across the levels of feeding rate were not the same for both species. Green sturgeon were generally less tolerant to feed restriction compared to white sturgeon. Conversely, at an optimal feeding rate, specific growth rate and feed efficiency were greater for green compared to white sturgeon. These results align well with [87] documenting that green sturgeon grow faster and larger than white sturgeon at early life stages.

### Synergistic effects of nutrition and salinity changes on growth performance of juvenile sturgeon

Overall, increasing the feeding rate in green sturgeon had a strong negative effect on muscle moisture content, likely causing a more subtle negative effect of muscle moisture on condition factor. The GAM analysis showed that the latter was a nonlinear decrease. The direct response of muscle moisture to feeding rate is in line with recent research in green sturgeon [48, 88], but a possible indirect effect on CF mediated by muscle moisture was first quantified. Juvenile green sturgeon with lower nutritional status (12.5, 25% of OFR) have been shown to have greater muscle moisture, regardless of salinity level [48]. In the current study, juvenile white sturgeon muscle moisture did not show a significant relationship to CF. Regardless of feeding rate and salinity factors, the data also showed noticeable inherent differences between the two species in terms of body composition, with juvenile white sturgeon having greater lipid and lower moisture. Greater lipid proportion supported the energy requirements of osmoregulatory process [89], measured by muscle water content in this case, without growth performance being affected.

This study demonstrated a mechanism by which an acute exposure to salinity affected growth performance of juvenile green sturgeon but may have not influenced white sturgeon growth performance. The strongest effect of salinity augment was a spike in PCNKA activity, which in turn led to a subtle negative effect on CF in green sturgeon (Fig 6). The GAM analysis showed that the latter was a nonlinear decrease, especially marked following a certain value of ceca enzyme activity. Although an increment in PCNKA activity following salinity exposure, regardless of feeding rate, was documented before [48] the likely indirect effect on CF mediated by PCNKA was first quantified in the present study. Broadly, an increase in the activity of this enzyme is characteristic of many euryhaline species following salinity changes [90–91], indicating good acclimatization to a new ambient salinity [41, 92]. Examining single-variable responses, most authors highlight the capacity of green sturgeon to respond to salinity challenges which is well established at a relatively young age [38, 40, 59, 88]. This study showed that this ability of juvenile green sturgeon to respond to unpredictable salinity fluctuations, such as those predicted to occur in SFBD with a changing global climate, occur at the cost of loss of growth performance. In white sturgeon, salinity exposure duration affected significantly the growth performance, adding to the effect of feeding rate.

Juvenile green and white sturgeon will likely be affected differently by forecasted larger and less stable salinity regimes coupled with shifts in prey abundance in the SFBD. Although the salinity exposure lasted for five days and did not simulate the biological consequences of long-term hyperosmotic exposure, the analysis enabled insights into comparing the mechanisms underlying the tolerance of the two species following both short and long-term nutrition and salinity changes. Three major trends can be anticipated for the growth performance (as measured by CF) of green and white sturgeon in the sensitive juvenile stage in the SFBD: (i) a decrease in prey abundance will be highly detrimental for the growth of both species; (ii) an acute increase in salinity within the limits studied can be tolerated by both species but the energy spent in osmoregulation may affect green sturgeon growth within the time window assessed; (iii) the effects of nutrition and salinity changes will be more complex in green sturgeon, with CF responding nonlinearly to interactions of salinity and nutrition status or time of salinity exposure. Green sturgeon would merit further scientific investigation to offset the effects of feed restriction and salinity as environmental stressors in the SFBD.

## References

[1] Sokolova IM. Energy-Limited Tolerance to Stress as a Conceptual Framework to Integrate the Effects of Multiple Stressors. Integr Comp Biol. 2013; 53: 597–608. 10.1093/icb/ict028 23615362

[2] Beatty SJ, Morgan DL, Lymbery AJ. Implications of climate change for potamodromous fishes. Glob Chang Biol. 2014; 20: 1794–1807. 10.1111/gcb.12444 24307662

[3] IPCC (Intergovernmental Panel on Climate Change). Working Group I Contribution to the IPCC Fifth Assessment Report Climate Change 2013: The Physical Sciences Basis Summary for Policymakers. Cambridge, UK: Cambridge University Press; 2013.

[4] Meehl GA, Washington WM, Collins WD, Arblaster JM, Hu A, Buja LE, et al How Much More Global Warming and Sea Level Rise? Science. 2005; 307: 1769–1772. 15774757 10.1126/science.1106663

[5] Cloern JE, Jassby AD. Drivers of change in estuarine-coastal ecosystems: Discoveries from four decades of study in San Francisco Bay. Rev Geophys. 2012; 50: RG4001.

[6] Fields PA, Graham JB, Rosenblatt RH, Somero GN. Effects of expected global climate change on marine faunas. Trends Ecol Evol. 1993; 8: 361–367. 10.1016/0169-5347(93)90220-J 21236196

[7] Roessig J, Woodley C, Cech J Jr, Hansen L. Effects of global climate change on marine and estuarine fishes and fisheries. Rev. Fish Biol Fish. 2004; 14: 251–275.

[8] Parmesan C. Ecological and Evolutionary Responses to Recent Climate Change. Annu Rev Ecol Evol Syst. 2006; 37: 637–669.

[9] Pörtner HO, Knust R. Climate change affects marine fishes through the oxygen limitation of thermal tolerance. Science. 2007; 315: 95–97. 17204649 10.1126/science.1135471

[10] NMFS (National Marine Fisheries Service). Endangered and threatened wildlife and plants: proposed threatened status for Southern distinct population segment of North American green sturgeon. Fed Reg. 2006; 71:17757–17766.

[11] CNDDB (California Natural Diversity Database). Database: California Natural Diversity Database at California Department of Fish and Game; 2009. Available: http://www.dfg.ca.gov/biogeodata/cnddb/

[12] Lee SY. Structural Equation Modeling: A Bayesian Approach. Chichester, UK: Wiley; 2007.

[13] Clough Y. A generalized approach to modeling and estimating indirect effects in ecology. Ecology. 2012; 93: 1809–1815. 22928410 10.1890/11-1899.1

[14] Bizzi S, Surridge BWJ, Lerner DN. Structural Equation Modelling: a novel statistical framework for exploring the spatial distribution of benthic macroinvertebrates in riverine ecosystems. River Res Appl. 2013; 29: 743–759.

[15] Moyle PB. Inland fishes of California Berkeley: University of California Press; 2002.

[16] Knowles N. Natural and management influences on freshwater inflows and salinity in the San Francisco Estuary at monthly to interannual scales. Water Resour Res. 2002; 38: 1289 10.1029/2001WR000360

[17] Cloern JE, Jassby AD, Thompson JK, Hieb KA. A cold phase of the East Pacific triggers new phytoplankton blooms in San Francisco Bay. Proc Natl Acad Sci U S A. 2007; 104: 18561–18565. 18000053 10.1073/pnas.0706151104 PMC2141816

[18] Knowles N, Cayan DR. Potential effects of global warming on the Sacramento/San Joaquin watershed and the San Francisco estuary. Geophys Res Lett. 2002; 29: 1891.

[19] Knowles N, Cayan D. Elevational Dependence of Projected Hydrologic Changes in the San Francisco Estuary and Watershed. Clim Change. 2004; 62: 319–336.

[20] Cayan D, Maurer E, Dettinger M, Tyree M, Hayhoe K. Climate change scenarios for the California region. Clim Change. 2008; 87: 21–42.

[21] Cloern JE, Knowles N, Brown LR, Cayan D, Dettinger MD, Morgan TL, et al. Projected Evolution of California's San Francisco Bay-Delta-River System in a Century of Climate Change. PLoS ONE. 2011; 6: e24465 10.1371/journal.pone.0024465 21957451 PMC3177826

[22] Boyce DG, Lewis MR, Worm B. Global phytoplankton decline over the past century. Nature. 2010; 466: 591–596. 10.1038/nature09268 20671703

[23] Winder M, Schindler DE. Climate change uncouples trophic interactions in an aquatic ecosystem. Ecology. 2004; 85: 2100–2106.

[24] Linville RG, Luoma SN, Cutter L, Cutter GA. Increased selenium threat as a result of invasion of the exotic bivalve Potamocorbula amurensis into the San Francisco Bay-Delta. Aquat Toxicol. 2002; 57: 51–64. 11879938 10.1016/s0166-445x(01)00265-x

[25] Cloern JE, Schraga TS, Lopez CB, Knowles N, Grover Labiosa R, Dugdale R. Climate anomalies generate an exceptional dinoflagellate bloom in San Francisco Bay. Geophys Res Lett. 2005; 32: L14608.

[26] Auad G, Miller A, Di Lorenzo E. Long-term forecast of oceanic conditions off California and their biological implications. J Geophys Res. 2006; 111: C09008 10.1029/2005JC003219 20411040

[27] Kogut NJ. Overbite clams, *Corbula amurensis*, defecated alive by white sturgeon, *Acipenser transmontanus* . Calif Fish Game. 2008; 94: 143–149.

[28] Silvestre F, Linares-Casenave J, Doroshov SI, Kültz D. A proteomic analysis of green and white sturgeon larvae exposed to heat stress and selenium. Sci Total Environ. 2010; 408: 3176–3188. 10.1016/j.scitotenv.2010.04.005 20435339 PMC3478132

[29] Schulte PM. What is environmental stress? Insights from fish living in a variable environment. J Exp Biol. 2014; 217: 23–34. 10.1242/jeb.089722 24353201

[30] Hamdoun A, Epel D. Embryo stability and vulnerability in an always changing world. Proc Natl Acad Sci U S A. 2007; 104: 1745–1750. 17264211 10.1073/pnas.0610108104 PMC1794293

[31] Stefansson SO, Imsland AK, Handeland SO. Food-deprivation, compensatory growth and hydro-mineral balance in Atlantic salmon (*Salmo salar*) post-smolts in sea water. Aquaculture. 2009; 290: 243–249. 10.1016/j.aquaculture.2009.02.024

[32] Imsland AK, Vage KA, Handeland SO, Stefansson SO. Growth and osmoregulation in Atlantic salmon (*Salmo salar*) smolts in response to different feeding frequencies and salinities. Aquac Res. 2011; 42: 469–479.

[33] Triebenbach SP, Smoker WW, Beckman BR, Focht R. Compensatory Growth after Winter Food Deprivation in Hatchery-Produced Coho Salmon and Chinook Salmon Smolts. N Am J Aquac. 2009; 71: 384–399.

[34] Mcenroe M, Cech JJ. Osmoregulation in juvenile and adult white sturgeon, Acipenser transmontanus. Environ Biol Fishes. 1985; 14: 23–30.

[35] Martinez-Alvarez RM, Hidalgo MC, Domezain A, Morales AE, Garcia-Gallego M, Sanz A. Physiological changes of sturgeon *Acipenser naccarii* caused by increasing environmental salinity. J Exp Biol. 2002; 205: 3699–3706. 12409496 10.1242/jeb.205.23.3699

[36] Jarvis PL, Ballantyne JS. Metabolic responses to salinity acclimation in juvenile shortnose sturgeon *Acipenser brevirostrum* . Aquaculture. 2003; 219: 891–909.

[37] Krayushkina LS, Semenova OG, Vyushina AV. Level of serum cortisol and Na^+^/K^+^ ATP-ase activity of gills and kidneys in different acipenserids. J Appl Ichthyol. 2006; 22: 182–187.

[38] Allen PJ, Cech JJ, Kultz D. Mechanisms of seawater acclimation in a primitive, anadromous fish, the green sturgeon. J Comp Physiol B. 2009; 179: 903–920. 10.1007/s00360-009-0372-2 19517116 PMC2745624

[39] Amiri BM, Baker DW, Morgan JD, Brauner CJ. Size dependent early salinity tolerance in two sizes of juvenile white sturgeon, *Acipenser transmontanus* . Aquaculture; 2009; 286: 121–126.

[40] Sardella B, Kültz D. Osmo- and ionoregulatory responses of green sturgeon (*Acipenser medirostris*) to salinity acclimation. J Comp Physiol B. 2009; 179: 383–390. 10.1007/s00360-008-0321-5 19066909

[41] Sardella BA, Kultz D. The Physiological Responses of Green Sturgeon (*Acipenser medirostris*) to Potential Global Climate Change Stressors. Physiol Biochem Zool. 2014; 87: 456–463. 10.1086/675494 24769709

[42] Krayushkina LS, Dyubin VP. The reaction of juvenile sturgeon to alteration of environmental salinity. Journal of Ichthyology. 1974; 14: 971–977.

[43] Jenkins WE, Smith TIJ, Heyward LD, Knott DM. Tolerance of shortnose sturgeon, *Acipenser brevirostrum*, to different salinity and dissolved oxygen concentrations. Proceedings of the Annual Conference of the Southeastern Association of Fish and Wildlife Agencies. 1993; 47: 476–484.

[44] Cataldi E, Barzaghi C, Di Marco P, Boglione C, Dini L, McKenzie DJ, et al Some aspects of osmotic and ionic regulation in Adriatic sturgeon *Acipenser naccarii*. I: Ontogenesis of salinity tolerance. J Appl Ichthyol. 1999; 15: 57–60.

[45] Jarvis PL, Ballantyne JS, Hogans WE. The influences of salinity on the growth of juvenile shortnose sturgeon. N Am J Aquac. 2001; 63: 272–276.

[46] McKenzie DJ, Cataldi E, Romano P, Taylor EW, Cataudella S, Bronzi P. Effects of acclimation to brackish water on tolerance of salinity challenge to young-of-the-year Adriatic sturgeon (*Acipenser naccarii*). Can J Fish Aquat Sci. 2001; 58: 1113–1121.

[47] Singer TD, Ballantyne JS. Sturgeon and paddlefish metabolism In: LeBreton GTO, Beamish FWH, McKinley RS, editors. Sturgeons and paddlefish of North America. Dordrecht: Kluwer Academic Publishers; 2002 pp. 167–194.

[48] Haller LY, Hung SSO, Lee S, Fadel JG, Lee J-H, Mcenroe M, et al The Effect of Nutritional Status on the Osmoregulation of Green Sturgeon (*Acipenser medirostris*). Physiol Biochem Zool. 2015; 88: 22–42. 10.1086/679519 25590591

[49] Cooke SJ, Suski CD. Ecological Restoration and Physiology: An Overdue Integration. BioScience. 2008; 58: 957–968.

[50] Van Eenennaam JP, Linares-Casenave J, Muguet J-B, Doroshov SI. Induced Spawning, Artificial Fertilization, and Egg Incubation Techniques for Green Sturgeon. N Am J Aquac. 2008; 70: 434–445.

[51] Van Eenennaam JP, Linares-Casenave J, Doroshov SI. Tank spawning of first generation domestic green sturgeon. J Appl Ichthyol. 2012; 28: 505–511.

[52] Cui Y, Hung SSO, Zhu X. Effect of ration and body size on the energy budget of juvenile white sturgeon. J Fish Biol. 1996; 49: 863–876.

[53] Lee S, Wang Y, Hung SSO, Strathe AB, Fangue NA, Fadel JG. Development of optimum feeding rate model for white sturgeon (*Acipenser transmontanus*). Aquaculture. 2014; 433: 411–420.

[54] Cui YB, Hung SSO, Deng DF, Yang YX. Growth performance of juvenile white sturgeon as affected by feeding regimen. Progressive Fish-Culturist. 1997; 59: 31–35.

[55] Cui Y, Hung SSO. A Prototype Feeding-Growth Table for White Sturgeon. Journal of Applied Aquaculture. 1996; 5: 25–34.

[56] Jones CE. Animal feed In: Williams S, editors. Official methods of analysis of the association of official analytical chemists. 14th ed. Arlington, VA, USA: Association of Official Analytical Chemists; 1984 pp. 152–160.

[57] Adams PB, Grimes C, Hightower JE, Lindley ST, Moser ML, Parsley MJ. Population status of North American green sturgeon, *Acipenser medirostris* . Environ Biol Fishes. 2007; 79: 339–356.

[58] Israel J, Drauch A, Gingras M. Life history conceptual model for white sturgeon. Sacramento, CA: Report to Bay Delta Ecosystem Restoration and Improvement Program; 2009. Available: https://nrm.dfg.ca.gov/FileHandler.ashx?DocumentID=28423

[59] Allen PJ, Mcenroe M, Forostyan T, Cole S, Nicholl MM, Hodge B, et al Ontogeny of salinity tolerance and evidence for seawater-entry preparation in juvenile green sturgeon, *Acipenser medirostris* . J Comp Physiol B. 2011; 181: 1045–1062. 10.1007/s00360-011-0592-0 21630040

[60] Quinn GP, Keough MJ. Experimental Design and Data Analysis for Biologists. Cambridge: Cambridge University Press; 2002.

[61] Sokal RR, Rohlf FJ. Introduction to Biostatistics. 2nd ed. New York: Dover Publications; 2009.

[62] Wheeler RE. lmPerm: Permutation tests for linear models; 2010. Available: http://CRAN.R-project.org/package=lmPerm

[63] Froese R. Cube law, condition factor and weight-length relationships: history, meta-analysis and recommendations. J Appl Ichthyol. 2006; 22: 241–253.

[64] Sarkar UK, Khan GE, Dabas A, Pathak AK, Mir JI, Rebello SC, et al Length weight relationship and condition factor of selected freshwater fish species found in River Ganga, Gomti and Rapti, India. J Environ Biol. 2013; 34: 951–956. 24558811

[65] Wood SN. Generalized Additive Models: An Introduction with R. Boca Raton: Chapman and Hall/CRC; 2006

[66] Zuur AF, Ieno EN, Walker N, Saveliev AA, Smith GM. Mixed Effects Models and Extensions in Ecology with R. New York: Springer; 2009.

[67] Zuur AF, Ieno EN, Elphick CS. A protocol for data exploration to avoid common statistical problems. Methods Ecol Evol. 2010; 1: 3–14.

[68] R Development Core Team. R: A Language and Environment for Statistical Computing R Foundation for Statistical Computing. Vienna, Austria: R Development Core Team; 2009 Available: http://www.r-project.org

[69] Wood SN. On p-values for smooth components of an extended generalized additive model. Biometrika. 2013; 100: 221–228

[70] Wood SN. A simple test for random effects in regression models. Biometrika. 2013; 100: 1005–1010.

[71] Arbuckle JL. IBM SPSS Amos 19 User’s Guide. Chicago, IL: Amos Development Corporation; 2010.

[72] Byrne BM. Structural Equation Modeling With AMOS: Basic Concepts, Applications, and Programming. 2nd ed. New York, London: Routledge Taylor & Francis Group; 2010.

[73] Browne MW. Asymptotically distribution-free methods for the analysis of covariance structures. Br J Math Stat Psychol. 1984; 37: 62–83. 6733054 10.1111/j.2044-8317.1984.tb00789.x

[74] Pörtner HO, Farrell AP. Physiology and climate change. Science. 2008; 322: 690–692. 10.1126/science.1163156 18974339

[75] Widdicombe S, Spicer JI. Predicting the impact of ocean acidification on benthic biodiversity: What can animal physiology tell us? J Exp Mar Bio Ecol. 2008; 366: 187–197.

[76] Hofmann GE, Todgham AE. Living in the Now: Physiological Mechanisms to Tolerate a Rapidly Changing Environment. Annu Rev Physiol. 2010; 72: 127–145. 10.1146/annurev-physiol-021909-135900 20148670

[77] Somero GN. The physiology of climate change: how potentials for acclimatization and genetic adaptation will determine ‘winners’ and ‘losers’. J Exp Biol. 2010; 213: 912–920. 10.1242/jeb.037473 20190116

[78] Pörtner H-O. Oxygen- and capacity-limitation of thermal tolerance: a matrix for integrating climate-related stressor effects in marine ecosystems. J Exp Biol. 2010; 213: 881–893. 10.1242/jeb.037523 20190113

[79] O’Donnell M, Hammond L, Hofmann G. Predicted impact of ocean acidification on a marine invertebrate: elevated CO2 alters response to thermal stress in sea urchin larvae. Mar Biol. 2009; 156: 439–446.

[80] Todgham AE, Hofmann GE. Transcriptomic response of sea urchin larvae *Strongylocentrotus purpuratus* to CO2-driven seawater acidification. J Exp Biol. 2009; 212: 2579–2594. 10.1242/jeb.032540 19648403

[81] Hofmann GE, Barry JP, Edmunds PJ, Gates RD, Hutchins DA, Klinger T, et al 2010. The Effect of Ocean Acidification on Calcifying Organisms in Marine Ecosystems: An Organism-to-Ecosystem Perspective. Annu Rev Ecol Evol Syst. 2010; 41: 127–147.

[82] Hung SSO, Lutes PB, Conte FS, Storebakken T. Growth and feed-efficiency of white sturgeon (*Acipenser-transmontanus*) sub-yearlings at different feeding rates. Aquaculture. 1989; 80: 147–153.

[83] Hung SSO, Liu W, Li HB, Storebakken T, Cui YB. Effect of starvation on some morphological and biochemical parameters in white sturgeon, Acipenser transmontanus. Aquaculture. 1997; 151: 357–363.

[84] Deng DF, Koshio S, Yokoyama S, Bai SC, Shao QJ, Cui YB, et al Effects of feeding rate on growth performance of white sturgeon (*Acipenser transmontanus*) larvae. Aquaculture. 2003; 217: 589–598.

[85] Yarmohammadi M, Shabani A, Pourkazemi M, Soltanloo H, Imanpour MR. Effect of starvation and re-feeding on growth performance and content of plasma lipids, glucose and insulin in cultured juvenile Persian sturgeon (*Acipenser persicus* Borodin, 1897). J Appl Ichthyol. 2012; 28: 692–696.

[86] De Riu N, Zheng KK, Lee JW, Lee SH, Bai SC, Moniello G, et al Effects of feeding rates on growth performances of white sturgeon (*Acipenser transmontanus*) fries. Aquac Nutr. 2012; 18: 290–296.

[87] Deng X, Van Eenennaam J, Doroshov S. Comparison of early life stages and growth of green and white sturgeon. In: Van Winkle W, Anders P, Secor D, Dixon D, editors. Biology, management, and protection of North American sturgeon. Bethesda, Maryland: American Fisheries Society Symposium 28; 2002. pp. 237–248

[88] Allen P, Cech JJ. Age/size effects on juvenile green sturgeon, *Acipenser medirostris*, oxygen consumption, growth, and osmoregulation in saline environments. Environ Biol Fishes. 2007; 79: 211–229.

[89] Polakof S, Arjona F, Sangiao-Alvarellos S, Martín Del Río M, Mancera J, Soengas J. Food deprivation alters osmoregulatory and metabolic responses to salinity acclimation in gilthead sea bream *Sparus auratus* . J Comp Physiol B. 2006; 176: 441–452. 16432730 10.1007/s00360-006-0065-z

[90] Jensen MK, Madsen SS, Kristiansen K. Osmoregulation and salinity effects on the expression and activity of Na+,K+-ATPase in the gills of European sea bass, *Dicentrarchus labrax* (L.). J Exp Zool. 1998; 282: 290–300. 9755480 10.1002/(sici)1097-010x(19981015)282:3<290::aid-jez2>3.0.co;2-h

[91] Rodríguez A, Gallardo MA, Gisbert E, Santilari S, Ibarz A, Sánchez J, et al Osmoregulation in juvenile Siberian sturgeon (*Acipenser baerii*). Fish Physiol Biochem. 2002; 26: 345–354.

[92] Imsland AK, Gunnarsson S, Foss A, Stefansson SO. Gill Na+, K+-ATPase activity, plasma chloride and osmolality in juvenile turbot (*Scophthalmus maximus*) reared at different temperatures and salinities. Aquaculture. 2003; 218: 671–683.

